# Monitoring of Allograft Adaptation After Kidney Transplantation in Pediatric Patients by Targeted Plasma Metabolomics

**DOI:** 10.3390/ijms26189190

**Published:** 2025-09-20

**Authors:** Jelena Klawitter, Bruce E. Kirkpatrick, Ryan Shillingburg, Jost Klawitter, Garrett Wheeler, Touraj Shokati, Melissa A. Cadnapaphornchai, Jeffrey L. Galinkin, Joshua M. Thurman, Uwe Christians

**Affiliations:** 1 Department of Anesthesiology, University of Colorado, Aurora, CO 80045, USAuwe.christians@cuanschutz.edu (U.C.); 2 Division of Renal Diseases and Hypertension, University of Colorado School of Medicine, Aurora, CO 80045, USA; 3 Emergency Department, University of Tennessee Medical Center, Knoxville, TN 37920, USA; 4 HepQuant, LLC, Denver, CO 80237, USA; 5 Division of Nephrology, Children’s Hospital of Philadelphia, Philadelphia, PA 19104, USA; 6 US Anesthesia Partners of Colorado, Greenwood Village, CO 80111, USA

**Keywords:** kidney transplant, metabolomics, kidney failure, pediatric patients, tacrolimus

## Abstract

End-stage kidney disease is preferably treated by kidney transplantation. The function of the allograft often determines kidney-controlled processes and requires long-term monitoring. Kidneys are organs with a very high metabolic rate, and, thus, a metabolomics approach is suitable to observe systemic metabolic changes that are related to graft adaptation. To understand these ongoing changes in post-transplant pediatric patients, we applied a targeted liquid chromatography/tandem mass spectrometry-based metabolomics approach. Time-dependent changes of 140 metabolites in plasma samples prospectively collected from 23 pediatric kidney graft recipients receiving tacrolimus-based immunosuppression were monitored over the first 4 years after transplantation and compared to levels prior to transplantation. Furthermore, by comparing the pre-transplant metabolite levels to those measured in healthy children, we were able to obtain insights into the pathways associated with kidney failure. Arginine biosynthesis, alanine, aspartate, glutamine, and glutamate metabolism, taurine and tryptophan metabolism were the most affected pathways that separate the pediatric patients with and without kidney failure. Accumulation of uremic toxins such as various tryptophan/kynurenine and tryptophan/indole metabolism pathway intermediates, and betaine and methionine cycle metabolites was evident in patients with restricted kidney function. Furthermore, reduced nicotinamide production, insufficient hydroxylation of phenylalanine to tyrosine, lowered cysteine, arginine, glutamine, taurine, and overall amino acid utilization, as well as diminished levels of protective antioxidants such as glutathione and vitamins B6 and C, were all the result of progressive kidney failure leading to transplantation. Importantly, following kidney transplantation and recovery of kidney function, the levels of most of the previously described metabolites normalized toward the levels observed in healthy participants. The here identified metabolic patterns could be used as markers to monitor the progression of pediatric chronic kidney disease patients towards kidney failure, and assuming their direct association with kidney function, they could serve as markers of successful graft adaptation.

## 1. Introduction

Since its first successful performance in 1954, kidney transplantation has become the most widespread organ engrafting procedure and the preferred treatment for those with kidney failure [1].

The introduction of the calcineurin inhibitors (CNIs) cyclosporine (CsA) in the 1980s [2] and tacrolimus (TAC) in the early 1990s [3] further revolutionized transplantation medicine. Currently, more than 95% of kidney transplant recipients are discharged with a CNI-based immunosuppressive regimen [4]. In pediatric kidney transplant recipients, the most common maintenance immunosuppression regimens at hospital discharge are based on TAC, mycophenolate mofetil (MMF), and steroids in 59.5%, followed by TAC and MMF reported by 33.2% of recipients [4]. Although advances in immunosuppressive protocols have reduced the incidence of acute rejection over the years [5], the long-term outcome of the kidney allograft remains largely unchanged due to the persistence of chronic allograft dysfunction [6]. In general, the estimated half-life for transplanted kidneys in children is 12–15 years; therefore, children with kidney failure often require more than one kidney transplant in their lifetime.

The long-term success of a kidney transplant depends on pretransplant donor–recipient serology matching as well as the ability to monitor transplant recipients and responsively adjust their medications, especially in case of allograft acute rejection episodes. Unfortunately, we are still relying on the measurement of serum creatinine levels and proteinuria to assess kidney function [7,8,9]. Thus, follow-up biopsies, both inconvenient to the patient and associated with hospitalization and variable histopathological analysis, are required to reach a definitive diagnosis [10,11,12].

The use of “omics” techniques that allow for the detection of multiple cellular products and that are able to bypass the limitations of the current routine diagnostic tools may lead to new diagnostic strategies aiding in the management of kidney transplant patients. Metabolomics can perform an unbiased and dynamic analysis of metabolites, thus making it an ideal candidate for the discovery of new markers of short- and long-term kidney graft function in the transplant patients [13,14,15,16,17]. Metabolite alterations accompany the progression of chronic kidney allograft dysfunction, and this may be relevant for the outcome, both in terms of graft survival and the health of the patient.

It was the goal of the present study to assess the time-dependent impact of a kidney transplant on plasma metabolite patterns in pediatric patients as surrogate markers for the normalization of pathological metabolic and physiological processes caused by kidney failure leading to transplantation. The plasma metabolome was evaluated using liquid chromatography-tandem mass spectrometry (LC-MS/MS) to gain insights into: (a) the metabolomic abnormalities evident in individuals with failing kidneys (namely in pediatric patients before the transplant procedure as compared to healthy children); (b) the short- and long-term changes in metabolite patterns and pathways affected by the kidney allograft during the first 4 years following transplantation in comparison to before transplantation in the same patient and a cohort of healthy children.

## 2. Results

### 2.1. Patient Characteristics

Plasma samples from 10 children and young adults were available at the start of the study, namely at baseline (prior to kidney transplant), and from 23 patients at pre-dose (within 7 days post-transplant and just before the initiation of the tacrolimus drug regimen) (Table 1). From these 23 patients, 15 and 10 patients continued to 1-year and 2-year follow-up visits, respectively (Table 1). Three patients were not available for follow-up visits (one withdrew, one could not be reached, and one patient moved away), whereas three patients experienced acute rejection episodes (biopsy-proven) after 3, 6, and 17 months, respectively. One patient with the earliest acute rejection episode was placed on a higher tacrolimus dose and kept in the study; another two were placed on another immunosuppressive regimen and did not return for follow-up visits. In seven additional patients, tacrolimus was discontinued primarily due to tremors and skeletomuscular problems, and in one case, due to the onset of symptomatic BK virus infection. Due to patients’ reluctance to participate, glomerular filtration rate (mGFR) was measured in only 15 patients (at 3 months post-transplant) using radiolabeled sodium iothalamate (Glofil^-125^); their median GFR normalized to plasma volume was 74.9 [63.3; 91.0] mL/min/1.73 m^2^. However, we were still able to calculate kidney function through the modified 2009 Schwartz equation. Directly after transplant (within 7 days, pre-dose), four out of 23 children still had elevated serum creatinine (1.4 to 9.8 mg/dL) and therefore low eGFR. The kidney function significantly improved towards 1 and 3 months, with children showing stable eGFR (>60 mL/min/1.73 m^2^). After the first year, two children presented with slightly lower eGFR of 50 and 58 mL/min/1.73 m^2^, respectively, and one of them, who continued to the 2-year follow-up, further declined to 47 mL/min/1.73 m^2^.

In addition to the transplant patients, we included samples from 98 healthy children in our analysis, and the characteristics of this control group, albeit limited, are summarized in Table 1.

### 2.2. Comparison of Metabolic Profiles of Children with Kidney Failure (Pre-Transplant Baseline Timepoint) with Healthy Children

**Figure 1:**
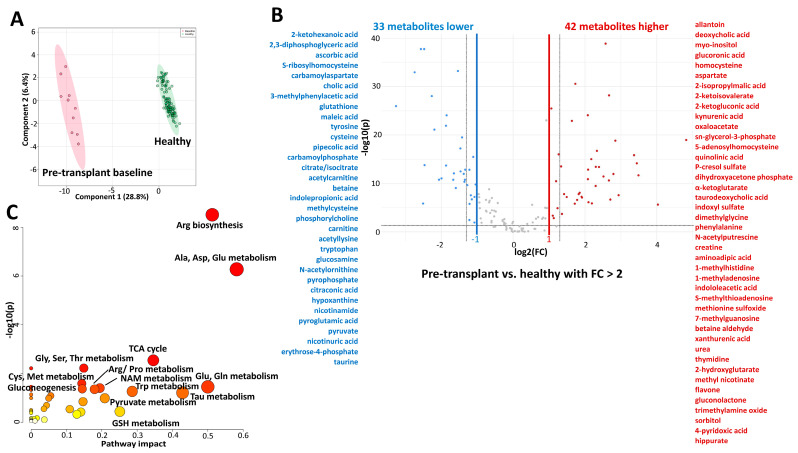
Differences between plasma metabolic profiles of children and young adults with kidney failure and healthy children. (**A**) The plot depicts separation of pediatric patients with kidney failure (pre-transplant, baseline samples, N = 10) and healthy participants (N = 98) utilizing the first two components of the partial least squares discriminant analysis (PLS-DA). Colored oval areas represent 95% confidence intervals of the respective groups. (**B**) Volcano plot revealed that 42 metabolites were increased (in red) and 33 decreased (in blue) in children with kidney failure versus healthy children, with a *p*-value FDR < 0.05 and a fold change > 2. X-axis corresponds to log2(Fold Change) and Y-axis to −log10(*p*-value). (**C**) Pathway analysis utilizing the referenced significantly changed 75 metabolites. The pathway impact on the *y*-axis from 0 (low impact) to 1 (strong impact) represents the values from the pathway topology analysis. Each circle denotes a pathway, and the fill color represents the significance of disturbances in that pathway from white (low significance) to red (higher significance). The relative peak areas were log-transformed and Pareto-scaled (mean-centered and divided by the square root of the standard deviation of each variable). All data was analyzed using the MetaboAnalyst 5.0 software. Abbreviations: Ala: alanine; Arg: arginine; Asp: aspartate; Cys: cysteine; Gln: glutamine; Glu: glutamate; Gly: glycine; GSH: glutathione; Met: methionine; NAM: nicotinamide; Phe: phenylalanine; Pro: proline; Ser: serine; Tau: taurine; Thr: threonine; Trp; tryptophan; Tyr: tyrosine.

The first step was to assess the extent of changes in the plasma metabolome in children with kidney failure shortly before transplantation in comparison to healthy children. At the pre-transplant baseline visit, we had access to plasma samples from 10 patients. As expected, PLS-DA revealed a strong separation between the metabolic profiles of children with kidney failure (N = 10) and healthy children (N = 98) (Figure 1A). Eighty-six metabolites were significantly different by more than 40% between the two groups (Table 2), with most metabolites changed by more than a factor of 2 (FDR < 0.05, Figure 1B).

The pathway enrichment analysis performed on the above-mentioned 86 compounds showed arginine biosynthesis, alanine, aspartate, glutamine, and glutamate metabolism, taurine metabolism, TCA cycle, and tryptophan metabolism to be the most affected pathways and the main separators between the metabolic profiles of children with kidney failure and healthy children (Figure 1C). Many of the known uremic toxins including (a) tryptophan/kynurenine metabolism pathway intermediates kynurenic acid, kynurenine, quinolinic acid and xanthurenic acid; (b) betaine metabolism pathway intermediates betaine aldehyde, dimethylglycine, and trimethylamine oxide (TMAO); (c) methionine cycle metabolites methionine sulfoxide, homocysteine and S-adenosylhomocysteine (SAH); as well as allantoin and hippurate were increased in children with kidney failure as compared to healthy children (Table 2, Figure 1B). In contrast, cytoprotective metabolites (a) tryptophan and nicotinamide; (b) betaine and taurine; (c) glutathione and its precursor cysteine, and methylcysteine were lower in children with kidney failure versus healthy children (Table 2, Figure 1B). Additional biomarker analysis using receiver operating characteristic (ROC) curves revealed fifty-six metabolites with area under the curve (AUC) values of above 0.90 and, thus, a good sensitivity potential for distinguishing healthy children from children with kidney failure and vice versa (Table 2).

In the next step, we assessed the impact of the transplanted kidney on the plasma metabolome within the first days after transplantation.

### 2.3. Comparison of Metabolic Profiles of Post-Transplant (Pre-Dose Timepoint) with Healthy Children

Plasma metabolic profiles of transplant patients before the first tacrolimus dose (N = 23, within 7 days post-transplant) were compared to those of the healthy participants (N = 98). Groups were still clearly separated by PLS-DA (Supplementary Figure S1A). Ninety-two metabolites were significantly different between the two groups by more than 40% (Supplementary Table S1), and eighty-one metabolites differed by more than two-fold (FDR < 0.05, Supplementary Figure S1B). Eleven metabolites were identified as newly changed (as they were not changed in pre-transplant patients); these included augmented levels of 2,3-dihydroxybenzoic acid, alanine, asparagine, glutamine, glycerophosphocholine, glycine, N-acetylglucosamine, proline, sarcosine, serine, and threonine (Table 1, Supplementary Table S2). On the other hand, eight metabolites that were different between the children at baseline (pre-transplant) and healthy children, including acetylphosphate, cysteine, gluconic acid, myo-inositol, orotate, phenylpropiolic acid, pyruvate, and succinate, normalized between the pre- and post-transplant timepoints and were no longer different between healthy and transplanted children at the pre-dose timepoint (Table 1, Supplementary Table S2).

### 2.4. Pre-Transplant (Baseline) Versus Post-Transplant (Pre-Dose)

Comparison between plasma metabolic profiles of patients at pre-transplant baseline and post-transplant pre-dose timepoints (paired, N = 10) revealed only a subtle separation between the two groups (PLS-DA, Supplementary Figure S2A). Sixteen metabolites were significantly higher and six lower in post-transplant pre-dose versus pre-transplant baseline samples (N = 10 paired, FDR < 0.05, fold change > 1.5, Supplementary Figure S2B). Seven of these twenty-two metabolites, which were higher in post-transplant samples, were amino acids involved in protein synthesis, and four that were lower were uremic toxins: 3-methylphenylacetic acid, allantoate, S-adenosylhomocysteine, and quinolinic acid (Supplementary Figure S2B).

Finally, the longer-term impact of the transplant kidney on the plasma metabolome was evaluated during the first 4 months, during the first year, and then up to 4 years after transplantation.

### 2.5. Changes Within the First Month Post Kidney Transplant and Start of Tacrolimus Regimen

The first 12 h following the start of the tacrolimus regimen (N = 17 at pre-dose and at 4 h, 8 h, and 12 h, respectively) did not induce further significant metabolic changes.

**Figure 2:**
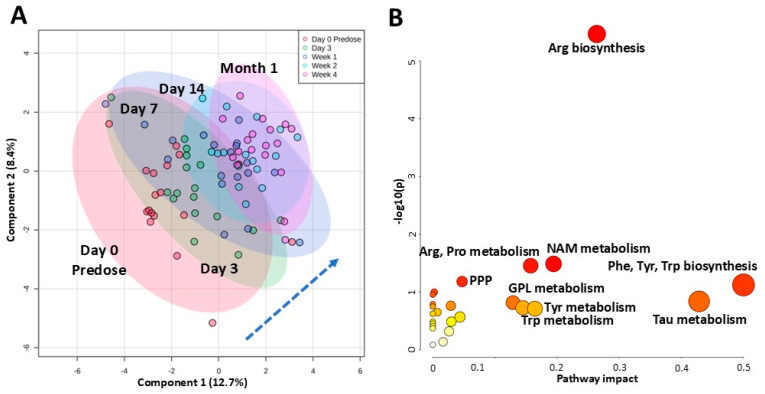
Time-dependent changes in plasma metabolic profiles in pediatric patients within the first month after kidney transplant. All patients received tacrolimus within 5 days of transplant. (**A**) The plot depicts time-dependent separation within transplant patients (N = 15–19) utilizing the first two components of the partial least squares discriminant analysis (PLS-DA). Colored oval areas represent 95% confidence intervals of the respective groups. (**B**) Pathway analysis of metabolites that significantly changed within the first month. Analysis was based on 30 metabolites that were identified as significantly changed with time (N = 15, paired repeated measures one-way ANOVA FDR < 0.05, with percentage change between pre-dose and 4 weeks at least 40%). The pathway impact on the *y*-axis from 0 (low impact) to 1 (strong impact) represents the values from the pathway topology analysis. Each circle denotes a pathway, and the fill color represents the significance of disturbances in that pathway from white (low significance) to red (higher significance). The relative peak areas were log-transformed and Pareto-scaled (mean-centered and divided by the square root of the standard deviation of each variable). Pathway analysis utilized the *KEGG* database. All data was analyzed using the MetaboAnalyst 5.0 software. Abbreviations: Arg: arginine; GPL: glycerophospholipid; NAM: nicotinamide; Phe: phenylalanine; PPP: pentose phosphate pathway; Pro: proline; Tau: taurine; Trp: tryptophan; Tyr: tyrosine.

Within the next month, however, metabolic profiles (N = 16–19 per timepoint) started to diverge, with 4-week plasma profiles moving furthest away from those obtained at pre-dose (Figure 2A). Nine metabolites were significantly different between the first five investigated timepoints: pre-dose, 3 days, 7 days, 14 days, and 4 weeks (one-way ANOVA in combination with Tukey’s post hoc test, FDR < 0.05). These included gluconic acid, N-acetylglutamate, N-acetylornithine, phenyllactic and quinolinic acids (levels declined from pre-dose to 4 weeks), as well as dihydroxybenzoic acid, carbamoylphosphate and taurine (levels increased from pre-dose to 4 weeks) (Supplementary Figure S3).

Within individual patients that had samples available at most of the first five timepoints (pre-dose, 3 days, 7 days, 14 days, and 4 weeks; N = 15), paired repeated measures one-way ANOVA revealed significant changes in 31 metabolites (FDR < 0.05, with percent change between pre-dose and 4 weeks at least 40%) (Table 3). From these 30 metabolites, 5 belonged to the arginine biosynthesis pathway (carbamoylphosphate, fumarate, N-acetylglutamate, N-acetylornithine, ornithine) and 4 to the tryptophan and nicotinamide metabolism pathways (anthranilic acid, nicotinamide, quinolinic acid, tryptophan) (Figure 2B).

### 2.6. Changes Within the First-Year Post Kidney Transplant

**Figure 3:**
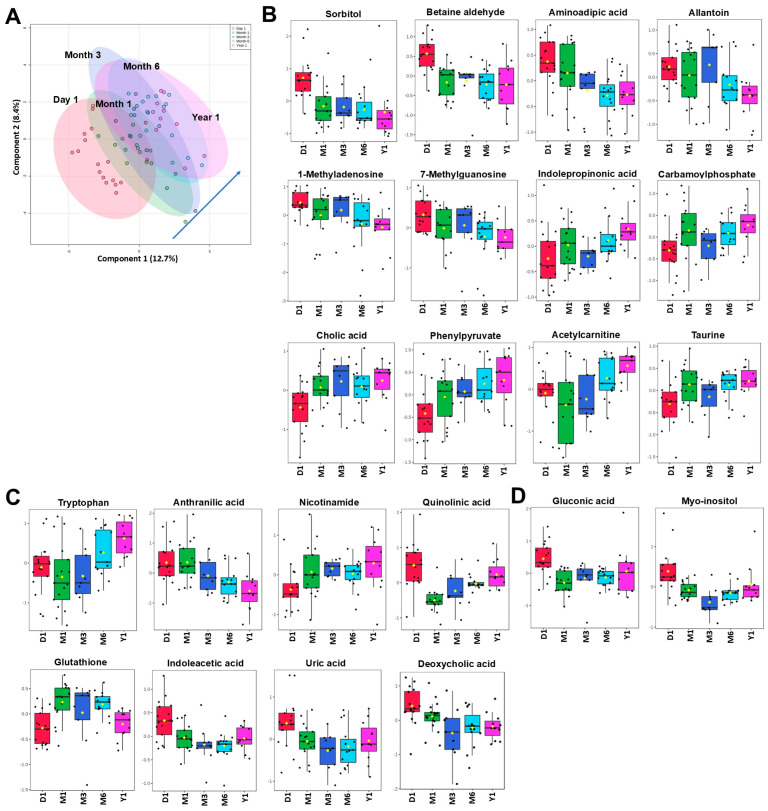
Time-dependent changes in plasma metabolic profiles in pediatric patients within the first year after kidney transplant. All patients received tacrolimus within 5 days of transplant. (**A**) The plot depicts time-dependent separation of transplant patients (N = 15–19) utilizing the first two components of the partial least squares discriminant analysis (PLS-DA). Colored oval areas represent 95% confidence intervals of the respective groups. (**B**–**D**) Time-dependent metabolite changes in plasma of pediatric kidney transplant patients. One-way ANOVA with FDR < 0.05 was used for statistical analysis. Data is presented as Box and Whisker plots. The lines in the boxes present the median (50th percentile), the boxes the 25th and 75th percentile (the lower and upper quartiles), and the whiskers the minimum and maximum values. The relative peak areas were log-transformed and Pareto-scaled (mean-centered and divided by the square root of the standard deviation of each variable). Yellow diamonds represent means of a metabolite for a specific group. Pathway analysis utilized the *KEGG* database. All data was analyzed using the MetaboAnalyst 5.0 software.

**Figure 4:**
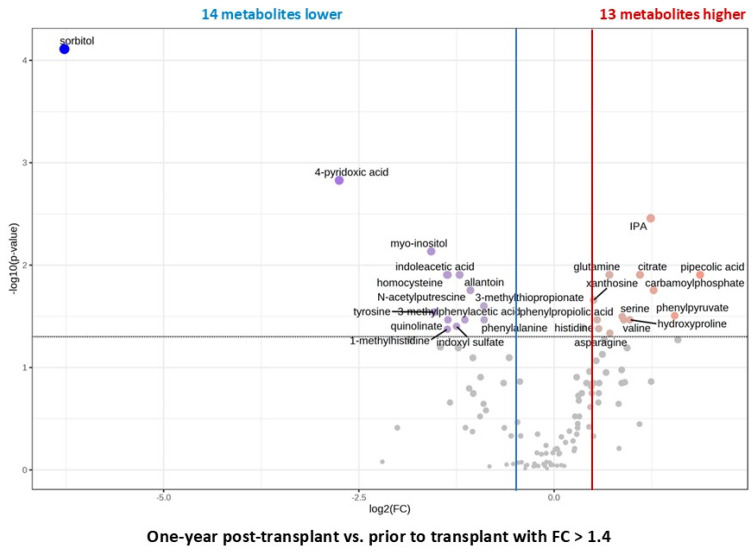
Changes between plasma metabolites of pediatric patients at pre-transplant and one-year post-kidney transplant timepoints. All patients received tacrolimus within 5 days after transplantation. Volcano plot revealed an increase in 14 plasma metabolites (in red) and a decrease in 14 metabolites (in blue) between pre-transplant and one-year post kidney transplant timepoints with a *p*-value FDR < 0.05 and a fold change of more than 40%. X-axis corresponds to log2(Fold Change) and Y-axis to −log10(*p*-value). The relative peak areas were log-transformed and Pareto-scaled (mean-centered and divided by the square root of the standard deviation of each variable). All data was analyzed using the MetaboAnalyst 5.0 software. Abbreviations: IPA: indolepropionic acid.

Within the first year, PLS-DA separated the metabolic profiles of pediatric kidney transplant patients (N = 15) (Figure 3A). Between the five investigated timepoints (12 h = Day 1, Month 1, Month 3, Month 6, and Year 1), 38 metabolites were identified as significantly changed by one-way ANOVA in combination with Tukey’s post hoc test, FDR < 0.05. Interestingly, levels of many of the uremic toxins continued to decline over time as they did within the first month post-surgery (Figure 3B,C). Other metabolites known to have cytoprotective properties increased with time (such as tryptophan, acetylcarnitine, hydroxyproline, taurine, indolepropionic acid) (Figure 3B–D). Overall, levels of most metabolites during the first year post-kidney transplant normalized towards the levels observed in healthy participants. Thirteen plasma metabolites, mainly highly accumulated uremic toxins, declined by more than 40% from pre- to post-kidney transplant timepoints (Figure 4; FDR < 0.05).

ROC analysis revealed a set of metabolites that was able to separate baseline (pretransplant) or pre-dose from 1-year timepoints with relatively high accuracy (AUC above 0.75). Twenty metabolites, such as allantoin, TMAO, acetylornithine, and carnitine, were shared between the comparisons (Supplementary Tables S3 and S4).

### 2.7. Changes Within 2 to 4 Years Post Kidney Transplant and Comparison to Healthy Subjects

**Figure 5:**
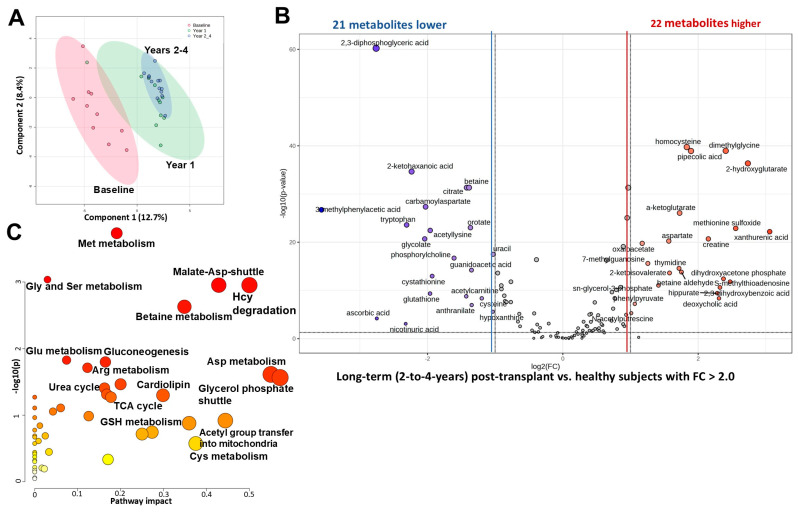
Time-dependent changes in plasma metabolic profiles in pediatric patients within the four years after kidney transplant. All patients received tacrolimus within 5 days of transplantation. (**A**) The plot depicts time-dependent separation of transplant patients (N = 10–13) utilizing the first two components of the partial least squares discriminant analysis (PLS-DA). Colored oval areas represent 95% confidence intervals of the respective groups. (**B**) Volcano plot revealed that 22 metabolites were increased (in red) and 21 decreased (in blue) in children with long-term kidney transplants (2 to 4 years) versus healthy children, with a *p*-value FDR < 0.05 and a fold change > 2. X-axis corresponds to log2(Fold Change) and Y-axis to −log10(*p*-value). (**C**) Pathway analysis utilizing the above-referenced significantly changed 43 metabolites. The pathway impact on the *y*-axis from 0 (low impact) to 1 (strong impact) represents the values from the pathway topology analysis. Each circle denotes a pathway, and the fill color represents the significance of disturbances in that pathway from white (low significance) to red (higher significance). The relative peak areas were log-transformed and Pareto-scaled (mean-centered and divided by the square root of the standard deviation of each variable). Pathway analysis utilized the *KEGG* database. All data was analyzed using the MetaboAnalyst 6.0 software.

Interestingly, combined metabolic plasma profiles of patients after 2 years (N = 10), 3 and 4 years (N = 3, respectively) were not different than those obtained after the first year (with no metabolites different among the timepoints) and did not separate from each other when compared to pre-transplant baseline (Figure 5A). None of the subjects with long-term follow-up experienced rejection episodes, so it could be speculated that the metabolic adaptations of the implanted kidney were largely complete after the first year.

Surprisingly, despite the observed normalization towards healthy subject levels, metabolic plasma profiles of children who received a kidney transplant 2-to-4 years prior remained different as compared to those of healthy children. Forty-three metabolites were still significantly changed by more than 2-fold between these two groups (FDR < 0.05, Figure 5B). Said 43 metabolites belong to metabolic pathways such as homocysteine degradation, malate-aspartate shuttle, methionine and urea cycle, glycine-, serine-, aspartate-, arginine-, proline-, glutamate-, and betaine metabolism, TCA cycle, glycerol phosphate shuttle, and gluconeogenesis.

Aspartate, oxaloacetate, and α-ketoglutarate are metabolites of the malate-aspartate shuttle, and all three were significantly lower in the plasma of transplanted versus healthy children. Moreover, the last two metabolites are also metabolites of the TCA cycle and gluconeogenesis pathways. Methionine metabolism intermediates, methionine sulfoxide, betaine aldehyde, dimethylglycine, and homocysteine were also still elevated, while cytoprotective metabolites within the same pathway, betaine, cysteine, and cystathionine, were still lower in children 2–4 years post-transplant (Figure 5B). Interestingly, within the tryptophan/kynurenine metabolism, most of the harmful intermediates previously identified as increased (pre-transplant) declined towards the healthy levels; with the exception of xanthurenic acid, which remained elevated, and tryptophan that was still lower in plasma 2–4 years after transplantation versus healthy children (Figure 5B).

## 3. Discussion

This study examined the differences between plasma metabolic profiles between children with kidney failure shortly before they received a graft and the longitudinal metabolic adaptations for up to four years after successful kidney transplantation in comparison to healthy children as a reference. All transplant patients received tacrolimus to prevent organ rejection. In addition to the assessment of the time-dependent changes in the plasma metabolome after kidney transplantation in children, the question was to what extent the transplanted kidney was able to “normalize” the plasma metabolome in the transplant children included in the study. Therefore, we also recruited 98 healthy children as a reference group.

After adjustment for multiple testing, ninety metabolites were significantly different by more than 40% between children with kidney failure and healthy children. Many of the identified metabolites are known uremic toxins, mainly identified in adults with kidney diseases, as specified by the European Uremic Toxin (EUTox) Work Group (https://database.uremic-toxins.org/soluteList.php, accessed on 1 September 2025). Two such uremic toxins, hippuric acid and sorbitol, accumulated in the blood of children with kidney insufficiency by more than 10-fold when compared to healthy children. Hippurate is generated from dietary polyphenols by the gut microbiota [19] and has been shown to positively correlate with the risk of kidney failure [20,21,22]. A recent serum metabolomics study performed in kidney transplant patients (N = 19) showed that the level of hippurate was more sensitive to any short-term change in kidney activity than creatinine [22]. Mechanistically, in animal models, hippurate has been shown to promote fibrosis, extracellular matrix imbalance, and oxidative stress [23]. Similarly, accumulation of sorbitol through an activated polyol pathway leads to proximal tubular cell dysfunction and injury [24]. Furthermore, in tissues without sorbitol dehydrogenase, such as the kidneys, accumulation of intracellular sorbitol draws fluid into the tissues, leading to elevated osmotic stress and fibrosis [25,26]. The third metabolite, which increased by more than 10-fold, was 4-pyridoxic acid, confirming previous reports in patients with chronic kidney disease (CKD) and kidney insufficiency [27,28]. While this degradation product of vitamin B6 accumulated, its active forms, pyridoxal-5′-phosphate and pyridoxal, were both reduced in children with kidney failure, showcasing the potential necessity for vitamin B6 supplementation in this patient population.

The metabolites that decreased the most in children with kidney failure were 2,3-diphosphoglycerate (2,3-DPG), ascorbic acid, glutathione and its precursor cysteine, Krebs cycle intermediates citrate and malate, just to name a few. 2,3-DPG is a phosphate ester that binds to deoxygenated hemoglobin and hence facilitates O_2_ delivery [29]. The observed decline in patients with kidney failure has been described previously [29]. It suggests the failure of said patients to produce it, despite their clear need for higher levels of 2,3-DPG so they can compensate for the underlying hypoxic conditions and decreased amount of oxygen available to tissues.

Interestingly, within the bile acid metabolism, deoxycholic and taurodeoxycholic acid levels were augmented in children with kidney failure. An increase in total bile acid production has been described in adult patients with ESKD versus healthy participants [30,31]. This increase is probably caused by abnormal enterohepatic circulation [32], abnormal reabsorption and secretion of bile acids by kidney tubuli and liver hepatocytes [33], and diminished excretion of bile acids due to low GFR [34].

The present metabolomics analysis revealed additional changes in uremic solutes, including those within the interlinked choline/betaine metabolism and methionine cycle, namely betaine aldehyde, dimethylglycine (DMG), trimethylamine oxide (TMAO), methionine sulfoxide, and S-adenosylhomocysteine (SAH) that accumulated in the blood of children with failing kidneys. Within the same pathway, the levels of betaine, a nutrient shown to have osmoprotective effects on the kidney [35], were reduced. Betaine serves as a vital methyl group donor in a transmethylation and detoxification process that catalyzes the conversion of homocysteine to methionine, with betaine itself being converted to DMG [36]. We also noted a deficiency in methionine cycle-related amino acid taurine in children with kidney failure versus healthy children, confirming previous reports [37,38].

Interestingly, several tryptophan-kynurenine pathway metabolites, including kynurenic acid, kynurenine, quinolinic acid, and xanthurenic acid, were elevated while tryptophan (TRP) and nicotinamide levels were lower in the blood of children with kidney failure, suggesting an activation of the kynurenine pathway (KP) in said patient population. The KP is the major catabolic pathway for TRP degradation, initiated through the conversion of TRP to kynurenine via indoleamine 2,3-dioxygenase (IDO). The KP plays an important role as the sole de novo nicotinamide adenine dinucleotide (NAD^+^) synthesis pathway in normal human physiology and functions as a counter-regulatory mechanism to mitigate immune responses during inflammation [39,40]. The observed activation of KP confirms the previous results obtained in adult patients with ESKD [39,41]. Kidney replacement therapy (hemodialysis and peritoneal dialysis) can decrease the circulating levels of KP metabolites to a certain degree [42]. However, higher IDO expression as well as activity (expressed as the increased kynurenine/tryptophan ratio) and oxidative stress were still seen in dialysis patients and were higher in patients undergoing peritoneal dialysis as compared to hemodialysis [42].

In addition to via the KP, TRP can be metabolized by gut microbiota via the indole pathway to indole and then further in the liver to indoxyl sulfate [43]. We have identified an increase in two metabolites within this pathway, namely indoleacetic acid (IAA) and indoxyl sulfate (IS), and a decline in plasma levels of indolepropionic acid (IPA). Recent studies have indicated that elevated serum IPA was correlated with a lower risk of rapid kidney function decline and developing CKD [44]. On the other hand, IAA and IS are known uremic toxins [45,46], shown to cause the development of tubulointerstitial fibrosis and glomerular sclerosis [47,48]. Accumulating evidence indicates that IAA, IS, p-cresyl sulfate (pCS), TMAO, and other toxins produced by the gut microbiota have a significant association with disease progression and outcomes in patients with CKD [47,48,49,50,51]. Limited animal and clinical studies have indicated that modulation of the gut microbiota could reduce uremic toxin production and delay CKD progression [52,53,54]. Thus, more studies are needed to establish the therapeutic efficacy of, for example, combined tryptophan, kynurenine, and indole metabolism modulation on CKD progression (potentially via reduction in IDO activity with concurrent supplementation with IPA). Successful kidney replacement and re-establishment of stable kidney function (>60 mL/min/1.73 m^2^) was able to normalize the level of microbiota-derived uremic toxins (IAA, IS, pCS, TMAO).

One of the unique metabolic functions of the kidney is phenylalanine hydroxylation to tyrosine, with the kidney shown to be the major donor of tyrosine to the systemic circulation [55]. In previous studies, it was shown that kidney failure leads to an impairment of whole-body phenylalanine hydroxylation, and our study confirmed elevation of plasma phenylalanine and a decline of tyrosine levels in children with kidney failure [56,57,58]. Tyrosine deficiency could lead to the limited availability of its products, such as neurotransmitters dopamine, catecholamines, and thyroid hormones, and thus tyrosine supplementation could limit an additional hormonal burden.

Immediately after kidney transplantation, plasma levels of several of the above-discussed uremic toxins, including 3-methylphenylacetic acid, allantoate, sorbitol and myo-inositol, S-adenosylhomocysteine and quinolinic acid, greatly declined and moved towards the levels in healthy participants. At the same time, levels of many previously restricted amino acids, such as glutamine, cysteine, valine, glycine, tyrosine, and serine, increased. Within the next month, further normalization of the metabolite concentrations and pathways formerly affected by the failing kidney was recorded. Levels of additional uremic toxins, including putrescine, TMAO, and phenylacetic acid, declined, whereas those of cytoprotective metabolites such as ornithine, hydroxyproline, and nicotinamide increased. Bile acid metabolism seemingly normalized as well, as evidenced by a reduction in deoxycholic acid levels. Similar pathways continued to further improve within the first 12 months post-kidney transplant. Overall, within the first year, the plasma metabolome seemed to stabilize with no significant change in the following years. Many of the highly accumulated uremic toxins were successfully cleared from circulation, while the production of protective metabolites improved.

Mechanistically, most metabolite biomarkers of kidney failure were markers of glomerular filtration, tubular function, or metabolites that reflect a decline in mitochondrial function, such as alterations in the methionine cycle, urea cycle, arginine synthesis, tryptophan-kynurenine, and indole pathways. Targeting these pathways by inhibition of IDO or supplementation of CKD patients with amino acids, antioxidants such as vitamins B6 and C, or IPA could provide effective treatment options for slowing down the progression of kidney disease and improving the allograft function and viability post-transplant.

Interestingly, some plasma metabolites remained different between transplanted and age-matched healthy children, with main differences still observed in the methionine cycle/trans-sulphuration pathway metabolites. The most studied of this pathway’s metabolites is homocysteine, a free radical producer and an independent risk factor for atherosclerotic disease [59]. Elevated levels of homocysteine are present in kidney transplant recipients and contribute to a high rate of both incident and recurrent cardiovascular disease (CVD) in said patients [60]. Confirming our findings, homocysteine levels have been shown to be elevated in children and adolescents with stable kidney transplants (2–7 years post-transplant [61,62]).

Betaine is an osmoprotective, anti-inflammatory metabolite and an active methyl donor within the methionine cycle [63,64]. It is produced from choline and betaine aldehyde via betaine aldehyde dehydrogenase (BADH). Betaine-homocysteine methyl transferase (BHMT) catalyzes the transfer of a methyl group from betaine to homocysteine while producing the uremic toxin dimethylglycine and methionine [63,64]. Reduced levels of betaine in the pediatric stable transplant patients were accompanied by higher betaine aldehyde, dimethylglycine, and, as aforementioned, increased homocysteine levels, suggesting an upregulation of BHMT and a reduction in BADH enzymatic activity.

One of the most common complications caused by tacrolimus is represented by de novo post-transplant diabetes mellitus (PTDM) [65,66,67,68,69]. Tacrolimus has been found to impair insulin secretion from pancreatic beta cells and increase insulin resistance, leading to a disruption in glucose metabolism [65,66,67,68,69,70]. In our study, none of the children who were followed up to 1- and 2-years developed PTDM; however, it must be noted that some of the changes identified in the TCA cycle and glycolysis metabolism could be a direct result of the tacrolimus treatment. Furthermore, a study performed in non-transplant patients treated with tacrolimus showed an increase in pipecolic acid and sarcosine, which we also have observed, and which could therefore be mediated by tacrolimus’ effects on cell metabolism [71].

Two to four years after transplantation, tryptophan-kynurenine metabolism was also still dysregulated in transplant patients, as evident by lower tryptophan and higher xanthurenic acid levels in plasma versus healthy subjects. As aforementioned, accumulation of kynurenine metabolites due to IDO overexpression is well described during acute and chronic kidney injury [40,72], and a higher xanthurenic acid (and resulting lower 3-hydroxykynurenine/xanthurenic acid ratio) has been shown to associate with the lower functional vitamin B6 status and long-term mortality in kidney transplant patients [73].

### Study Limitations

We acknowledge a relatively small pediatric kidney transplant patient population that was performed in a single center. However, the recruitment and retention of pediatric transplant patients in a prospective study with several years of follow-up is challenging. We recognize that the statistical power due to the small sample size might be low and we tried to partially control for it by applying low FDR < 0.05 and at least 40% change in effect. It is a known fact that exposure to immunosuppressive drugs related to non-immune and immune injuries can cause slow deterioration and premature failure of organ transplants. The present study, however, was not adequately powered to investigate the mechanisms underlying acute rejection, since only one patient who experienced a rejection episode remained in the study. Furthermore, it should be noted that, while the kidney function recovered to stable values (>60 within the first month post-transplantation) and remained stable at the 6-month timepoint, at 1 year, two patients experienced a slightly lower than normal eGFR. This decline in kidney function continued to progress in one patient who remained in the study at 2 years. The metabolic profiles of both subjects, however, did not separate from the other patients when subjected to PLSA-DA analysis and were therefore still included in the statistical analyses.

A future multi-center study consisting of a larger cohort size would help to strengthen statistical power and delineate the effects of alloimmune processes on metabolic profiles. The longitudinal nature and multiple collection timepoints, as in our study design, would help discover predictive signatures of rejection prior to clinical diagnosis and for monitoring health and functionality of the transplant kidney. All samples were stored at −80 °C and were analyzed in yearly batches. Previous studies have shown that only up to 2% of the plasma metabolome was altered within the first seven years of storage at −80 °C [74]; thus, we did not have concerns regarding the stability of plasma metabolites at ultra-low temperatures.

## 4. Materials and Methods

### 4.1. Trial Design

Participants were recruited through The Kidney Center at Children’s Hospital of Colorado in Aurora, CO, USA. The study protocol: “In Vivo Assessment of Calcineurin Inhibitor Toxicity in Children” was approved on 9 May 2014 (amendment approved on 2 June 2016) and monitored by the Colorado Multiple Institution Review Board (COMIRB Protocol 11-0799, Aurora, CO, USA). Written informed assent/consent was obtained. The study was monitored by COMIRB and adhered to the principles set forth by the Declaration of Helsinki and its amendments.

Briefly, children and young adults aged 1–21 years undergoing kidney transplant surgery with planned treatment with tacrolimus were enrolled in the study. Kidney diseases that necessitated or contributed to the organ transplant were various and are as follows: Fanconi syndrome, Alport syndrome, microscopic polyangiitis, multi-cystic dysplastic kidney, polycystic kidney disease, ischemic nephropathy due to diaphragmatic hernia, IgA nephropathy, obstructive nephropathy, reflux nephropathy, hydronephrosis, and congenital renal dysplasia. Exclusion criteria included treatment with CNI within 12 weeks preceding transplantation, discontinuation of previous CNI use due to non-kidney-related side effects, and pregnancy. Basic clinical parameters as well as sample collections (plasma and urine) were planned for the following timepoints: baseline (pre-transplant), pre-dose (post-transplant, prior to start of tacrolimus), 1 day, 3 days, 7 days, 2 weeks, 4 weeks, 3 months, 6 months, 1 year and yearly hereafter (for as long as possible). The initiation dose of tacrolimus ranged between 1 mg daily (6 patients), 2 mg (5 patients), 3 mg (5 patients), 4 mg (1 patient), 5 mg (1 patient), 6 mg (2 patients), and 8 mg daily (3 patients). Kidney function was assessed by mGFR using the GLOFIL-125 (sodium iothalamate I-125 injection) method after a single intravenous injection [75]. Thyroid blockade was performed prior to tracer administration, and research subjects were well hydrated. Following measurement of a background blood sample and tracer calibration, 30µCi (1.11 megabecquerels) of sodium iothalamate was injected intravenously. Certified nuclear technologists at the University of Colorado Hospital Nuclear Medicine department performed testing adhering to strict protocols. Due to many patients’ reluctance to participate in GLOFIL-125 measurements, we were able to collect mGFR in 15 patients at 3 months post-transplant and in 4 patients at 9 months post-transplant.

Modified 2009 Schwartz equation (eGFR = (0.413 × height)/serum creatinine) was used to calculate the estimated GFR (eGFR) at available timepoints [76]. Except for the additional research blood draws and urine collection, there were no additional study-related procedures or modifications of routine clinical practices.

The original study protocol aimed to identify the changes in plasma and urine metabolite patterns responsible for the negative effects of CNIs on kidney health in pediatric patients after kidney transplantation. However, as only 3 of the enrolled 23 patients experienced acute rejection episodes (with 2 patients subsequently placed on a different immunosuppressive regimen and lost to the study) and with most of the children maintaining satisfactory kidney function throughout the study, we were not adequately powered to address the original study aims. Therefore, the current paper aims to identify metabolic patterns that can be used as markers of successful graft adaptation.

In summary, twenty-three patients (followed for more than 2 weeks) were enrolled. Three patients missed the follow-up visits. All samples that were collected were utilized for the presented metabolomics studies.

### 4.2. Healthy Participants

The control cohort consisted of plasma samples collected from 98 fasting, healthy children undergoing minor dental, orthopedic, and ENT (ear, nose, and throat) surgical procedures. Sample collection was approved by COMIRB and occurred at the same clinical site (Children’s Hospital of Colorado). Upon plasma generation from ethylenediaminetetraacetic acid (EDTA) anticoagulated blood using a temperature-controlled centrifuge (+4 °C), all samples (from transplant patients and healthy participants) were stored at −80 °C within 4 h prior to analysis.

### 4.3. Targeted Metabolomics

Plasma samples were extracted according to a published protocol [77,78]. Briefly, samples were centrifuged and mixed with methanol to create an 80% (volume/volume) methanol solution and were incubated overnight at −80 °C to allow for protein precipitation. Following overnight incubation, samples were centrifuged, and supernatants were dried in a SpeedVac concentrator (Savant, ThermoFisher, Waltham, MA, USA). Samples were reconstituted with 20 µL water/methanol (80:20, volume/volume). Multiple reaction monitoring (MRM) using positive/negative ion-switching high-performance liquid chromatography-tandem mass spectrometry (5500 QTRAP HPLC-MS/MS, Sciex, Concord, ON, Canada [79]) was used for analysis. Of the 196 metabolites monitored, we were able to quantify 140 (please refer to Supplementary Materials for more details). Quality control samples were included for monitoring of intensity and retention time shifts.

Once the data were acquired, MultiQuant (v2.1.1, Sciex, Foster City, CA, USA) software was used for data processing. For between-sample normalization, the intensity values for each sample were summed, and the median value of the sums across all samples was determined.

### 4.4. Statistical Analysis

MetaboAnalyst (version 5.0 and 6.0; University of Alberta, Edmonton, AB, Canada) was used for statistical analysis of metabolomics data [80]. Analysis of changes in metabolites between healthy and transplant patients, as well as the analysis of time-related changes within transplanted children, was performed by utilizing the *t*-test (unpaired or paired) and ANOVA testing (with Tukey post hoc analysis). False discovery rate (FDR) < 0.05 was utilized to correct for type I errors in multiple testing. Partial Least-Squares Discriminant Analysis (PLS-DA) was utilized as a tool for assessing the discriminatory and predictive ability of metabolites and metabolite profiles for discrimination between subject groups. Relative peak intensities were log-transformed to reduce the influence of extreme values and to meet the homogeneity of variance assumption, and then normalized by Paretto-scaling (mean-centered and divided by the square root of the standard deviation of each variable). Pathway analysis was performed using the pathway analysis tool in MetaboAnalyst. This tool uses both pathway enrichment analysis based on the R-package—GlobalTest, analyzing compound concentration values, as well as on pathway topological analysis accounting for the impact of individual measured metabolites within the pathway. The goal of assessing pathway impact is to account for pathway structure and the intuitive concept that central or nodal positions in a pathway will have a greater impact than marginal or isolated positions. Total or maximal importance for each pathway is designated as 1, whereas the importance of measured metabolites to that pathway is designated as the cumulative percentage from matched metabolite nodes. Finally, the ability of metabolomic markers to serve as a measure of graft adaptation was tested using Receiver Operator Characteristic (ROC) curve analysis, with a *p* < 0.05 considered significant.

## 5. Conclusions

In summary, the present metabolomics study uncovered multiple metabolites and metabolic pathways that separated pediatric patients with kidney failure from healthy children and could therefore serve as biomarkers of the end-stage kidney disease in said patients. Furthermore, we showed that the plasma metabolic signature of stable kidney transplant patients changed over time towards normal but is still different from that of age-matched healthy children, probably at least partially due to an ongoing immune/inflammatory response or as a response to tacrolimus therapy. Based on the present results, it can be speculated that improving the identified metabolic deficiencies through, e.g., supplementation with betaine or vitamin B6 may potentially extend graft and recipient health. 

## Figures and Tables

**Table 1:** Patient characteristics at the pre-dose visit (N = 23, measurements taken within the first week following kidney transplant), 6 months (N = 18), one year, and two years (N = 10) visits (N = 15) following kidney transplant surgery/start of tacrolimus. One patient experienced an acute rejection episode after 3 months, was placed on a higher tacrolimus dose, and was included in the statistics for 6 and 12 months, respectively. An additional patient, included in the 2-year visit summary, has been placed on a sirolimus regimen after 19 months on tacrolimus due to symptomatic BK virus infection. Our patient population matches the characteristics of the overall pediatric kidney transplant population, with candidates aged 12 years and older and of white race reported as the largest proportion [18]. Limited demographics are presented for our healthy participants control group: age, gender, and race are available for all 98, whereas the results of the comprehensive metabolic panel (CMP; clinical analyzer) were available for a subset of 34 subjects. Values are presented as mean ± SD. * *p* < 0.05 for pre-dose visit versus 24 months post-transplant comparison. Abbreviations: BUN: blood urea nitrogen; SBP: systolic blood pressure; WBC: white blood cell count.

Characteristic	Healthy	Predose (N = 23)	1 Month (N = 15)	6 Months (N = 18)	12 Months (N = 15)	24 Months (N = 10)
Age (years)	12 ± 5	12 ± 4.3	11 ± 4.2	13 ± 4.1	12 ± 3.4	12 ± 2.7
% Female	53	48	60	39	33	30
Race % White	90	87	80	89	87	80
Race % Other	10	13	20	11	13	20
Height (cm)	-	138 ± 22	133 ± 21	140 ± 19	139 ± 18	137 ± 17
Weight (kg)	-	40 ± 19	36 ± 16	40 ± 14	40 ± 13	37 ± 13
eGFR	-	66 ± 30	79 ± 15	71 ± 19	78 ± 16	74 ± 22
Creatinine (mg/dL)	0.6 ± 0.1	1.5 ± 2.1	0.7 ± 0.2	0.9 ± 0.3	0.8 ± 0.2	0.8 ± 0.3
Hypertension, treated	-	N = 11	N = 6	N = 8	N = 7	N = 3
SBP (mmHg)	-	122 ± 12	113 ± 9	108 ± 15	111 ± 18	111 ± 8.6
BUN (mg/dL)	13 ± 4.3	29 ± 25	20 ± 5.6	18 ± 6.3	17 ± 6.6	19 ± 6.9
Bicarbonate (mmol/L)	24 ± 3.4	23 ± 2.5	23± 2.4	24 ± 3.1	23 ± 2.5	23 ± 1.9
Chloride (mmol/L)	104 ± 2.2	108 ± 4.5	107 ± 3.8	106 ± 2.6	106 ± 3.3	106 ± 2.0
Potassium (mmol/L)	4.2 ± 0.4	4.0 ± 0.5	4.3 ± 0.5	4.4 ± 0.5	4.2 ± 0.3	4.2 ± 0.3
Sodium (mmol/L)	141 ± 2.3	138 ± 2.8	139 ± 2.7	140 ± 2.4	140 ± 2.7	139 ± 2.2
Hematocrit (%)	42 ± 4.3 *	28 ± 3.4 *	35 ± 4.6	35 ± 6.8	37 ± 3.9	40 ± 4.7
Platelet (×10^9^/L)	-	210 ± 99	265 ± 43	289 ± 109	279 ± 63	278 ± 38
WBC (×10^9^/L)	6.2 ± 3.9	6.0 ± 2.7	8.0 ± 2.4	4.6 ± 2.0	5.8 ± 1.9	6.7 ± 2.1
Glucose (mg/dL)	93 ± 19	86 ± 11	86 ± 14	91 ± 10	90 ± 13	81 ± 8.5

**Table 2:** Plasma metabolite changes between children and young adults with kidney failure (at pre-transplant visit, N = 10) and healthy participants (N = 98). Metabolites with false discovery rate (FDR) < 0.05 and fold change of more than 40% between the groups are presented. The relative peak areas were log-transformed and Pareto-scaled (mean-centered and divided by the square root of the standard deviation of each variable). Data was analyzed using MetaboAnalyst 5.0 software.

	FDR	Fold Change
1-methyladenosine	1.34 × 10^−6^	4.57
1-methylhistidine	9.60 × 10^−16^	4.52
2,3-diphosphoglyceric acid	1.77 × 10^−38^	0.17
2-hydroxyglutarate	6.16 × 10^−29^	6.37
2-isopropylmalic acid	2.07 × 10^−4^	2.57
2-ketohexanoic acid	1.09 × 10^−33^	0.15
2-ketoisovalerate	1.56 × 10^−8^	2.67
3-methylphenylacetic acid	1.66 × 10^−11^	0.24
3-methylthiopropionate	1.02 × 10^−5^	1.86
4-pyridoxic acid	6.95 × 10^−15^	10.9
5-hydroxyindoleacetic acid	2.02 × 10^−3^	1.75
2-ketogluconic acid	6.48 × 10^−8^	2.81
7-methylguanosine	1.84 × 10^−17^	5.04
acetyllysine	1.09 × 10^−12^	0.39
α-ketoglutarate	8.63 × 10^−8^	3.78
allantoin	4.68 × 10^−4^	2.15
aminoadipic acid	7.82 × 10^−7^	4.28
arginine	7.01 × 10^−8^	0.52
argininosuccinate	1.63 × 10^−10^	0.48
ascorbic acid	1.43 × 10^−6^	0.18
aspartate	3.04 × 10^−14^	2.53
betaine	5.87 × 10^−34^	0.34
betaine aldehyde	3.99 × 10^−14^	5.24
carbamoylaspartate	8.98 × 10^−29^	0.21
carbamoylphosphate	1.71 × 10^−11^	0.31
carnitine	3.13 × 10^−20^	0.37
citraconic acid	1.46 × 10^−13^	0.43
citrate	1.77 × 10^−38^	0.18
creatine	1.37 × 10^−13^	4.25
cysteine	1.99 × 10^−14^	0.27
deoxycholic acid	1.48 × 10^−3^	2.19
dihydroxyacetone phosphate	5.54 × 10^−8^	3.75
dimethylglycine	8.01 × 10^−25^	4.23
erythrose-4-phosphate	6.08 × 10^−8^	0.47
gluconic acid	1.29 × 10^−5^	2.36
gluconolactone	1.45 × 10^−19^	7.19
glucosamine	1.42 × 10^−13^	0.40
glutamate	1.17 × 10^−5^	1.92
glutathione	9.14 × 10^−12^	0.25
glycolic acid	8.95 × 10^−7^	0.52
hippurate	2.38 × 10^−6^	16.3
homocysteine	9.63 × 10^−17^	2.41
indoleacetic acid	1.12 × 10^−11^	4.78
indolepropionic acid	2.68 × 10^−26^	0.36
indoxyl sulfate	1.57 × 10^−13^	4.10
kynurenic acid	1.55 × 10^−6^	3.29
kynurenine	1.17 × 10^−3^	1.79
maleic acid	1.18 × 10^−22^	0.27
methionine sulfoxide	3.68 × 10^−14^	4.93
methyl nicotinate	2.24 × 10^−7^	6.53
methylcysteine	6.06 × 10^−18^	0.36
myo-inositol	3.39 × 10^−11^	2.29
N-acetylcarnitine	9.10 × 10^−10^	0.33
N-acetylphosphate	1.26 × 10^−5^	0.53
N-acetylputrescine	1.32 × 10^−11^	4.23
nicotinamide	2.75 × 10^−8^	0.44
nicotinuric acid	1.21 × 10^−2^	0.47
orotate	9.23 × 10^−4^	1.62
oxaloacetate	2.63 × 10^−31^	3.32
p-cresol sulfate	2.04 × 10^−18^	3.71
phenylalanine	2.85 × 10^−10^	2.57
phenyllactic acid	1.95 × 10^−3^	1.56
phenylpropiolic acid	1.70 × 10^−3^	0.53
phosphorylcholine	3.28 × 10^−13^	0.36
p-hydroxybenzoate	1.58 × 10^−8^	0.51
pyroglutamic acid	1.99 × 10^−7^	0.45
pyridoxal	3.61 × 10^−3^	0.43
pyruvate	3.24 × 10^−16^	0.46
quinolinic acid	8.41 × 10^−9^	3.61
S-adenosylhomocysteine	1.58 × 10^−8^	3.54
shikimate	1.70 × 10^−3^	1.86
S-methylthioadenosine	3.42 × 10^−10^	4.92
sn-glycerol-3-phosphate	2.59 × 10^−7^	3.44
sorbitol	1.03 × 10^−19^	10.3
S-ribosylhomocysteine	1.62 × 10^−14^	0.18
succinate	1.19 × 10^−2^	1.46
taurine	4.50 × 10^−5^	0.59
taurodeoxycholic acid	3.89 × 10^−6^	3.89
thymidine	2.40 × 10^−11^	6.35
trimethylamine oxide	2.63 × 10^−8^	7.66
tryptophan	1.71 × 10^−11^	0.39
tyrosine	5.33 × 10^−14^	0.27
urea	1.25 × 10^−39^	5.96
uric acid	8.71 × 10^−24^	1.90
xanthurenic acid	3.71 × 10^−12^	5.72

**Table 3:** Metabolite changes in plasma of pediatric kidney transplant patients within the first month post-transplant. Adjusted *p*-values (paired repeated measures one-way ANOVA, (false discovery rate) FDR < 0.05) and fold change factors (4-week post-tacrolimus start to pre-dose, minimum of 40% change) within individual patients (N = 15) are presented. The normalized peak areas were log-transformed and Pareto-scaled. Data was analyzed using MetaboAnalyst 5.0 software.

	FDR	Fold Change 4-Week to Pre-Dose
putrescine	0.0141	0.25
acetylcarnitine	0.0141	0.29
N-acetylornithine	0.0141	0.31
quinolinic acid	0.0004	0.32
deoxycholic acid	0.0071	0.44
gluconic acid	0.0022	0.44
trimethylamine oxide	0.0124	0.45
N-acetylglutamate	0.0106	0.47
guanidoacetic acid	0.0141	0.52
N-acetylputrescine	0.0183	0.60
phenylalanine	0.0127	0.60
phenylacetic acid	0.0306	0.60
fumarate	0.0183	1.46
ornithine	0.0183	1.47
citraconic acid	0.0339	1.64
taurine	0.0287	1.67
2-ketohexanoic acid	0.0156	1.68
uridine	0.0141	1.73
proline	0.0172	1.74
carbamoylphosphate	0.0438	1.85
nicotinamide	0.0424	1.95
tyrosine	0.0296	1.99
glycerophosphocholine	0.0141	2.06
flavone	0.0172	2.17
sn-glycerol-3-phosphate	0.0141	2.18
anthranilic acid	0.0047	2.26
cholesteryl sulfate	0.0028	2.32
dihydroxybenzoic acid	0.0053	2.43
hypoxanthine	0.0382	2.44
pipecolic acid	0.0295	2.54

## Data Availability

The data presented in this study are available upon request from the corresponding author.

## Supplementary Materials

The following supporting information can be downloaded at: https://www.mdpi.com/article/10.3390/ijms26189190/s1.
