# Supplementary material for: Monitoring of Allograft Adaptation After Kidney Transplantation in Pediatric Patients by Targeted Plasma Metabolomics
Source: Int J Mol Sci. 2025 Sep 20;26(18):9190. doi: 10.3390/ijms26189190 (PMC12471270; doi:10.3390/ijms26189190)

Supplementary Materials

Monitoring of Allograft Adaptation After Kidney Transplantation in Pediatric Patients by Targeted Plasma Metabolomics

Jelena Klawitter [1,2,*], Bruce E. Kirkpatrick [1], Ryan Shillingburg [3], Jost Klawitter [1], Garrett Wheeler [1], Touraj Shokati [4], Melissa A. Cadnapaphornchai [5], Jeffrey L. Galinkin [6], Joshua M. Thurman [2] and Uwe Christians [1]

[1] Department of Anesthesiology, University of Colorado, Aurora, CO 80045, USA
[2] Division of Renal Diseases and Hypertension, University of Colorado School of Medicine, Aurora, CO 80045, USA
[3] Emergency Department, University of Tennessee Medical Center, Knoxville, TN 37920, USA
[4] HepQuant, LLC, Denver, CO 80237, USA
[5] Division of Nephrology, Children's Hospital of Philadelphia, Philadelphia, PA 19104, USA
[6] US Anesthesia Partners of Colorado, Greenwood Village, CO 80111, USA
* Correspondence: jelena.klawitter@cuanschutz.edu

Supplementary Table S1: Differences in plasma metabolites between pediatric transplant patients at predose (post-transplant, before tacrolimus start, N=23) and healthy children (N=98). Metabolites with false discovery rate (FDR) < 0.05 and fold change of more than 40% between the groups are presented. The relative peak areas were log-transformed and Paretto-scaled (mean-centered and divided by the square root of the standard deviation of each variable). Data was analyzed using MetaboAnalyst 5.0 software.

	FDR	Fold Change
**1-methyladenosine**	1.34E-06	4.57
**1-methylhistidine**	9.60E-16	4.52
**2,3-diphosphoglyceric acid**	1.77E-38	0.17
**2-hydroxyglutarate**	6.16E-29	6.37
**2-isopropylmalic acid**	2.07E-04	2.57
**2-ketohexanoic acid**	1.09E-33	0.15
**2-ketoisovalerate**	1.56E-08	2.67
**3-methylphenylacetic acid**	1.66E-11	0.24
**3-methylthiopropionate**	1.02E-05	1.86
**4-pyridoxic acid**	6.95E-15	10.9
**5-hydrodyindole acetic acid**	2.02E-03	1.75
**5-ketogluconic acid**	6.48E-08	2.81
**7-methylguanosine**	1.84E-17	5.04
**acetyllysine**	1.09E-12	0.39
**a-ketoglutarate**	8.63E-08	3.78
**allantoin**	4.68E-04	2.15
**aminoadipic acid**	7.82E-07	4.28
**arginine**	7.01E-08	0.52
**argininosuccinate**	1.63E-10	0.48
**ascorbic acid**	1.43E-06	0.18
**aspartate**	3.04E-14	2.53
**betaine**	5.87E-34	0.34
**betaine aldehyde**	3.99E-14	5.24
**carbamoylaspartate**	8.98E-29	0.21
**carbamoylphosphate**	1.71E-11	0.31
**carnitine**	3.13E-20	0.37
**citraconic acid**	1.46E-13	0.43
**citrate**	1.77E-38	0.18
**creatine**	1.37E-13	4.25
**creatinine**	3.06E-26	2.09

	FDR	Fold Change
**cysteine**	1.99E-14	0.27
**deoxycholic acid**	1.48E-03	2.19
**dihydroxyacetone phosphate**	5.54E-08	3.75
**dimethylglycine**	8.01E-25	4.23
**erythrose-4-phosphate**	6.08E-08	0.47
**flavone**	1.16E-12	6.93
**gluconic acid**	1.29E-05	2.36
**gluconolactone**	1.45E-19	7.19
**glucosamine**	1.42E-13	0.40
**glutamate**	1.17E-05	1.92
**glutathione**	9.14E-12	0.25
**glycolic acid**	8.95E-07	0.52
**hippuric acid**	2.38E-06	16.3
**hypoxanthine**	1.72E-06	0.43
**indole**	1.01E-26	0.10
**indoleacetic acid**	9.34E-09	3.93
**indolepropionic acid**	3.47E-15	0.41
**indoxyl sulfate**	3.65E-12	3.65
**kynurenic acid**	1.55E-06	3.29
**kynurenine**	1.17E-03	1.79
**maleic acid**	1.18E-22	0.27
**methionine sulfoxide**	3.68E-14	4.93
**methyl nicotinate**	2.24E-07	6.53
**methylcysteine**	6.06E-18	0.36
**myo-inositol**	3.39E-11	2.29
**N-acetylornithine**	2.29E-10	0.41
**N-acetylalanine**	9.63E-17	2.41
**N-acetylcarnitine**	9.10E-10	0.33
**N-acetylglutamate**	4.54E-11	0.39
**N-acetylphosphate**	1.26E-05	0.53
**N-acetylputrescine**	1.32E-11	4.23
**nicotinamide**	2.75E-08	0.44
**nicotinuric acid**	1.21E-02	0.47
**orotate**	9.23E-04	1.62
**oxaloacetate**	2.63E-31	3.32
**p-cresol sulfate**	7.53E-15	4.12
**phenylalanine**	4.28E-13	2.93
**phenyllactic acid**	1.95E-03	1.56
**phenylpropiolic acid**	1.70E-03	0.53
**phosphorylcholine**	3.28E-13	0.36

	FDR	Fold Change
**p-hydroxybenzoate**	1.58E-08	0.51
**pipecolic acid**	8.24E-25	0.28
**pyroglutamic acid**	1.99E-07	0.45
**pyrophosphate**	3.61E-03	0.43
**pyruvate**	3.24E-16	0.46
**quinolinic acid**	8.41E-09	3.61
**S-adenosylhomocysteine**	1.58E-08	3.54
**shikimate**	1.70E-03	1.86
**S-methylthioadenosine**	3.42E-10	4.92
**sn-glycerol-3-phosphate**	2.59E-07	3.44
**sorbitol**	1.03E-19	10.3
**S-ribosylhomocysteine**	1.62E-14	0.18
**succinate**	1.19E-02	1.46
**taurine**	4.50E-05	0.59
**tyrosine**	5.43E-14	0.25
**thymidine**	2.40E-11	6.35
**trimethylamine oxide**	2.63E-08	7.66
**tryptophan**	1.71E-11	0.39
**urea**	1.25E-39	5.96
**uric acid**	8.71E-24	1.90
**xanthurenic acid**	3.71E-12	5.72

Supplementary Table S2: Linear model analysis of intraindividual time-dependent changes (LIMMA) in plasma metabolic profiles of children and young adults within the first year after kidney transplant and start of tacrolimus. We had access to samples at pre-dose, week 1, month 1, month 6 and year 1 within individual patients (N=14). Significantly changed metabolites ($p<0.05$) as related to the pre-dose (T0) are presented. The relative peak areas were log-transformed and Paretto-scaled (mean-centered and divided by the square root of the standard deviation of each variable). All data was analyzed using time-series meta functional analysis module within the MetaboAnalyst 5.0 software.

	Day 7 to T0	Month 1 to T0	Month 6 to T0	Year 1 to T0	p-value
**indoleacetic acid**	**0.14**	**0.35**	**0.25**	**-0.23**	8.1E-04
**sn-glycerol-3-phosphate**	**0.32**	**0.54**	**0.00**	**-0.17**	2.1E-03
**glycerophosphocholine**	**0.36**	**0.53**	**0.06**	**-0.14**	2.2E-03
**anthranilic acid**	**0.36**	**0.39**	**-0.30**	**-0.62**	1.7E-08

**thymidine**	**0.30**	**0.52**	**-0.12**	**-0.15**	3.2E-03
**ornithine**	**0.28**	**0.17**	**-0.11**	**-0.24**	8.6E-03
**allantoin**	**0.07**	**0.15**	**-0.15**	**-0.42**	9.0E-03
**1-methyladenosine**	**0.08**	**0.11**	**-0.30**	**-0.48**	4.1E-02
**cysteine**	**0.11**	**0.03**	**-0.26**	**-0.34**	4.7E-02
**betaine aldehyde**	**0.06**	**-0.20**	**-0.18**	**-0.24**	2.3E-02
**deoxycholic acid**	**0.08**	**-0.21**	**-0.38**	**-0.26**	4.8E-02
**acetylcarnitine**	**-0.68**	**-0.73**	**-0.04**	**0.36**	4.5E-07
**S-ribosylhomocysteine**	**-0.29**	**-0.22**	**-0.46**	**0.05**	1.5E-02
**2,3-dihydroxybenzoic acid**	**0.32**	**0.74**	**0.71**	**0.68**	1.2E-04
**taurine**	**0.35**	**0.44**	**0.49**	**0.57**	7.0E-04
**pipecolic acid**	**0.43**	**0.48**	**0.77**	**0.63**	1.4E-03
**citrate/isocitrate**	**0.16**	**0.32**	**0.51**	**0.53**	2.4E-03
**carbamoylphosphate**	**0.53**	**0.50**	**0.51**	**0.63**	2.5E-03
**tryptophan**	**0.25**	**0.57**	**0.14**	**0.08**	3.9E-03
**phenylpyruvate**	**0.17**	**0.28**	**0.57**	**0.60**	6.4E-03
**nicotinamide**	**0.45**	**0.35**	**0.40**	**0.61**	6.8E-03
**hydroxyproline**	**0.27**	**0.14**	**0.36**	**0.47**	9.5E-03
**tyrosine**	**0.40**	**0.54**	**0.29**	**0.26**	1.8E-02
**aspartate**	**0.13**	**0.03**	**0.11**	**0.39**	3.1E-02
**arginine**	**0.20**	**0.36**	**0.13**	**0.34**	4.0E-02
**quinolinic acid**	**-0.69**	**-0.77**	**-0.37**	**-0.17**	1.1E-05
**indoxyl sulfate**	**-0.34**	**-0.36**	**-0.89**	**-0.86**	5.6E-07
**acetylornithine**	**-0.79**	**-0.76**	**-1.01**	**-0.53**	1.3E-05
**4-pyridoxic acid**	**-0.32**	**-0.16**	**-0.59**	**-0.61**	9.0E-05
**atrolactic acid**	**-0.52**	**-0.28**	**-0.17**	**-0.01**	1.1E-04
**guanidoacetic acid**	**-0.43**	**-0.57**	**-0.69**	**-0.63**	9.7E-04
**sorbitol**	**-0.55**	**-0.50**	**-0.83**	**-0.80**	1.0E-03
**N-acetylglutamate**	**-0.33**	**-0.55**	**-0.47**	**-0.55**	1.6E-03
**phenyllactic acid**	**-0.53**	**-0.45**	**-0.05**	**-0.21**	3.2E-03
**p-cresol sulfate**	**-0.41**	**-0.56**	**-0.46**	**-0.42**	4.3E-03
**hippurate**	**-0.26**	**-0.34**	**-0.38**	**-0.52**	6.0E-03
**N-acetylputrescine**	**-0.20**	**-0.33**	**-0.44**	**-0.41**	7.0E-03
**myo-inositol**	**-0.11**	**-0.38**	**-0.46**	**-0.29**	8.3E-03
**asparagine**	**-0.25**	**-0.29**	**-0.37**	**-0.18**	1.1E-02
**7-methylguanosine**	**-0.16**	**-0.11**	**-0.37**	**-0.46**	2.3E-02
**threonine**	**-0.19**	**-0.19**	**-0.39**	**-0.34**	2.9E-02

Supplementary Table S3: Receiver Operating Characteristic (ROC) analysis of plasma metabolic profiles obtained in children and young adults at pre-transplant baseline (N=10) and one year after kidney transplant (N=15). The relative peak areas obtained from metabolomic analysis were log-transformed and Paretto-scaled (mean-centered and divided by the square root of the standard deviation of each variable) and then subjected to univariate ROC analysis within the biomarker analysis tool of MetaboAnalyst 6.0 software. Abbreviations: AUC: area under the curve (cutoff of 0.75 was applied), FC: fold change with reference to baseline; TMAO: trimethylamine oxide.

Name	AUC	Ttest	log2FC
**4-pyridoxic acid**	0.99	2.30E-05	2.75
**sorbitol**	0.96	1.96E-05	4.15
**indolepropionic acid**	0.95	8.12E-05	-1.24
**citrate**	0.94	7.80E-04	-1.10
**allantoin**	0.93	9.52E-04	1.21
**indoleacetic acid**	0.91	6.82E-04	1.36
**myo-inositol**	0.90	2.84E-04	1.57
**carnitine**	0.90	8.23E-03	-0.37
**homocysteine**	0.89	1.06E-03	1.37
**3-methylthiopropionate**	0.89	2.92E-03	0.90
**glutamine**	0.86	1.05E-03	-0.71
**phenylalanine**	0.85	5.72E-03	0.90
**phenylpropiolic acid**	0.85	5.85E-03	-0.55
**3-methylphenylacetic acid**	0.85	6.10E-03	1.14
**xanthosine**	0.85	2.39E-03	-0.50
**serine**	0.85	4.44E-03	-0.87
**N-acetylputrescine**	0.85	1.77E-03	1.07
**tyrosine**	0.85	3.57E-03	1.53
**carbamoylphosphate**	0.84	1.77E-03	-1.27
**valine**	0.84	6.36E-03	-0.89
**hydroxyproline**	0.84	6.35E-03	-0.97
**quinolinic acid**	0.84	5.23E-03	1.36
**histidine**	0.83	8.74E-03	-0.57
**cysteine**	0.83	1.74E-02	-0.93
**1-methylhistidine**	0.83	9.17E-03	1.37
**TMAO**	0.83	1.29E-02	1.84
**nicotinamide**	0.82	1.33E-02	-1.58
**asparagine**	0.82	1.04E-02	-0.71
**serotonin**	0.82	2.60E-02	-0.54

Name	AUC	Ttest	log2FC
**phenylpyruvate**	0.81	4.10E-03	-1.54
**gluconolactone**	0.81	1.72E-02	1.23
**gluconate**	0.80	2.37E-02	1.04
**glycolate**	0.79	1.38E-01	0.87
**pyruvate**	0.79	1.30E-02	-0.64
**maleic acid**	0.79	3.27E-02	-0.86
**3-phosphoglycerate**	0.79	4.86E-02	-1.24
**indoxyl sulfate**	0.79	7.67E-03	1.25
**anthranilate**	0.78	4.20E-02	0.94
**acetylornithine**	0.78	1.61E-02	1.46
**glycine**	0.77	5.57E-02	-0.58
**sarcosine**	0.76	3.48E-02	-0.45
**aspartate**	0.75	4.24E-02	-0.29
**glucosamine**	0.75	3.64E-02	-0.66

Supplementary Table S4: Receiver Operating Characteristic (ROC) analysis of plasma metabolic profiles obtained in children and young adults at pre-dose (N=23) and one year after kidney transplant (N=15). The relative peak areas obtained from metabolomic analysis were log-transformed and Paretto-scaled (mean-centered and divided by the square root of the standard deviation of each variable) and then subjected to univariate ROC analysis within the biomarker analysis tool of MetaboAnalyst 6.0 software. Abbreviations: AUC: area under the curve (cutoff of 0.75 was applied), FC: fold change with reference to pre-dose; TMAO: trimethylamine oxide.

Name	AUC	Ttest	log2FC
**nicotinamide**	0.91	2.02E-04	-1.38
**allantoin**	0.90	1.12E-03	1.27
**taurine**	0.89	1.42E-04	-1.02
**acetylornithine**	0.88	1.40E-04	2.06
**citrate**	0.88	1.65E-02	-0.65
**sorbitol**	0.87	2.11E-04	2.76
**N-acetylglutamate**	0.87	5.04E-04	1.51
**3-methylthiopropionate**	0.86	1.18E-03	1.06
**7-methylguanosine**	0.85	1.20E-03	1.23
**carbamoylphosphate**	0.84	1.22E-03	-1.08
**gluconate**	0.83	5.64E-03	1.48
**pipecolic acid**	0.82	1.96E-03	-0.85

Name	AUC	Ttest	log2FC
**myo-inositol**	0.82	3.77E-03	0.86
**cystathionine**	0.82	1.43E-02	1.32
**4-pyridoxic acid**	0.81	6.39E-03	1.27
**aspartate**	0.80	5.85E-03	-0.52
**carnitine**	0.80	1.25E-02	-0.39
**glucose-6-phosphate**	0.80	3.40E-02	1.36
**acetylcarnitine**	0.80	9.87E-03	-0.81
**phenyllactic acid**	0.79	1.86E-02	0.76
**TMAO**	0.79	5.69E-02	1.40
**1-methylhistidine**	0.78	4.07E-02	1.35
**1-methyladenosine**	0.78	1.04E-02	2.08
**hydroxyproline**	0.77	8.96E-03	-0.85
**N-acetylputrescine**	0.77	2.97E-02	0.71
**maleic acid**	0.76	1.33E-02	-0.67
**anthranilate**	0.76	1.80E-02	2.14
**guanidoacetic acid**	0.76	6.81E-03	1.25
**cysteine**	0.76	1.01E-01	0.66
**pyroglutamic acid**	0.75	4.77E-02	0.65

Supplementary Figure S1. Differences between plasma metabolic profiles of healthy children and children after kidney transplant. *(A) The plot depicts separation of pediatric patients that obtained kidney transplant (within one week after transplant surgery but before the initiation of immunosuppressive treatment, transplant pre-dose, N=23) and healthy participants (N=98) utilizing the first two components of the partial least square discriminant analysis (PLS-DA).* Colored oval areas represent 95% confidence intervals of the respective groups. *(B) Volcano plot revealed that 41 metabolites were increased (in red) and 40 decreased (in blue) in said children, with a p-value FDR<0.05 and a fold change >2.* X-axis corresponds to log2(Fold Change) and Y-axis to −log10(p-value).

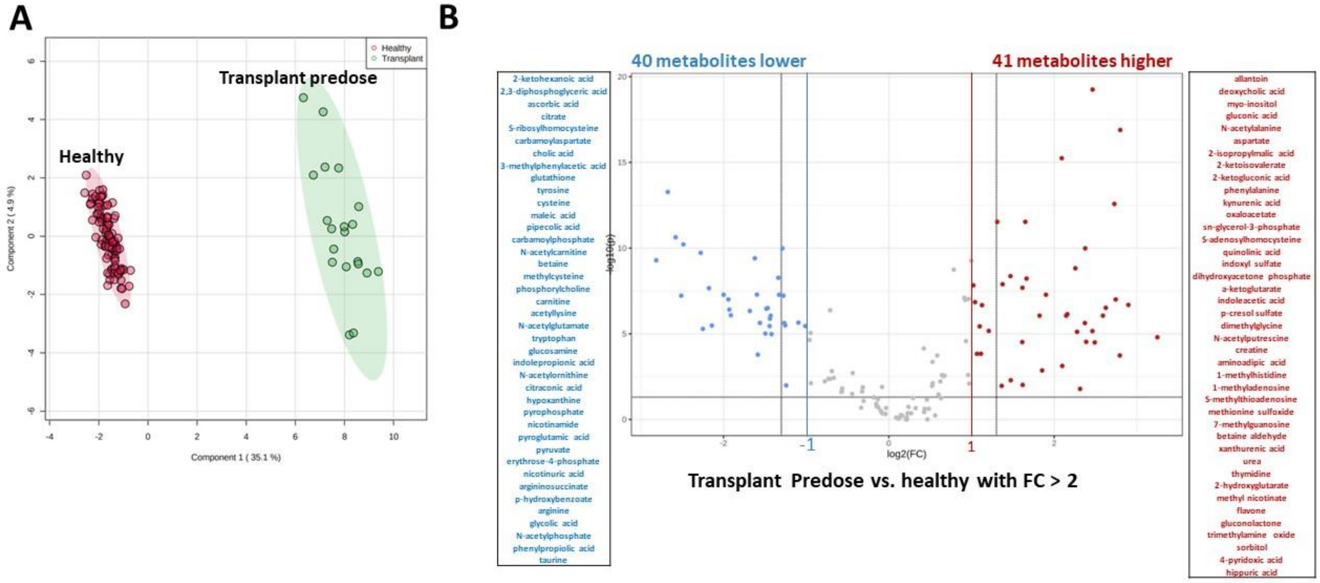

Supplementary Figure S2: Differences between plasma metabolic profiles of children shortly before and within the first week after kidney transplant. *(A) The plot depicts separation of the same pediatric patients at baseline (pre-transplant, N=10) and after kidney transplant (within one week after transplant surgery but before the initiation of immunosuppressive treatment. transplant pre-dose, N=10) utilizing the first two components of the partial least square discriminant analysis (PLS-DA).* Colored oval areas represent 95% confidence intervals of the respective groups. *(B) Volcano plot revealed that 6 metabolites were increased (in red) and 16 decreased (in blue) in said children, with a p-value FDR<0.05 and a fold change >1.5.* X-axis corresponds to log2(Fold Change) and Y-axis to −log10(p-value).

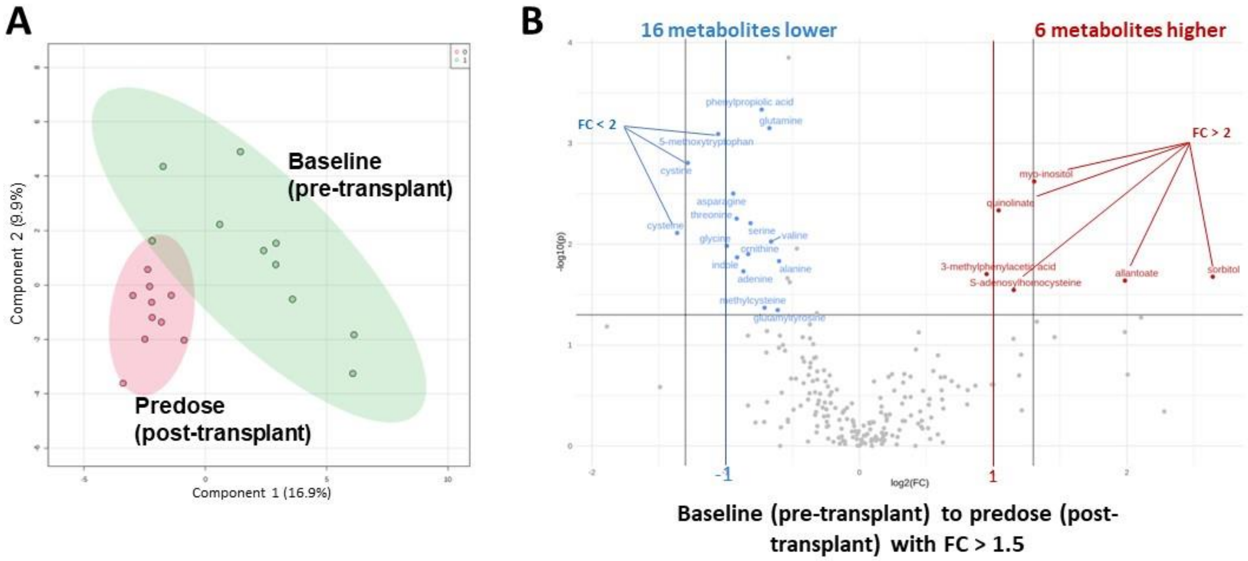

Supplementary Figure S3: Time-dependent changes of plasma metabolic profiles in pediatric patients within the first 4 weeks after kidney transplant. All patients received tacrolimus within 5 days of transplant. One-way ANOVA with FDR<0.05 was used for statistical analysis. Data is presented as Box and Whisker plots (N=16-19 per time point). The lines in the boxes present the median (50th percentile), the boxes the 25th and 75th percentile (the lower and upper quartiles) and the whiskers the minimum and maximum values. The relative peak areas of respective metabolites were log-transformed and Paretto-scaled (mean-centered and divided by the square root of the standard deviation of each variable).

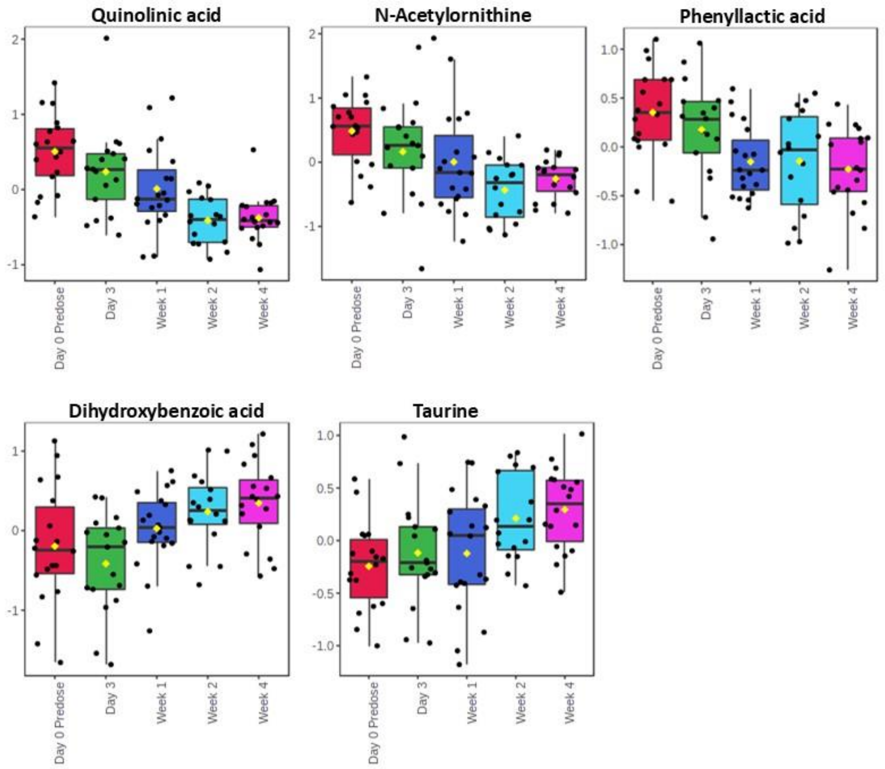

Supplement: Supplementary file 1 [file ijms-26-09190-s001.zip › ijms-3597183-supplementary.pdf]
